# α-Conotoxin Decontamination Protocol Evaluation: What Works and What Doesn’t

**DOI:** 10.3390/toxins9090281

**Published:** 2017-09-14

**Authors:** Matthew W. Turner, John R. Cort, Owen M. McDougal

**Affiliations:** 1 Biomolecular Sciences Graduate Programs, Boise State University, Boise 83725, ID, USA; matthewturner1@u.boisestate.edu; 2 Department of Chemistry and Biochemistry, Boise State University, Boise 83725, ID, USA; 3 Biological Sciences Division, Pacific Northwest National Laboratory, Richland 99354, WA, USA; John.Cort@pnnl.gov

**Keywords:** conotoxin, select agent, circular dichrosim (CD) spectroscopy, mass spectrometry (MS), high performance liquid chromatography (HPLC), peptide decontamination, biosafety protocols

## Abstract

Nine publically available biosafety protocols for safely handling conotoxin peptides were tested to evaluate their decontamination efficacy. Circular dichroism (CD) spectroscopy and mass spectrometry (MS) were used to assess the effect of each chemical treatment on the secondary and primary structure of α-CTx MII (L10V, E11A). Of the nine decontamination methods tested, treatment with 1% (*m*/*v*) solution of the enzymatic detergent Contrex™ EZ resulted in a 76.8% decrease in α-helical content as assessed by the mean residue ellipticity at 222 nm, and partial peptide digestion was demonstrated using high performance liquid chromatography mass spectrometry (HPLC-MS). Additionally, treatment with 6% sodium hypochlorite (*m*/*v*) resulted in 80.5% decrease in α-helical content and complete digestion of the peptide. The Contrex™ EZ treatment was repeated with three additional α-conotoxins (α-CTxs), α-CTxs LvIA, ImI and PeIA, which verified the decontamination method was reasonably robust. These results support the use of either 1% Contrex™ EZ solution or 6% sodium hypochlorite in biosafety protocols for the decontamination of α-CTxs in research laboratories.

## 1. Introduction

The venom of predatory marine snails from the genus *Conus* is comprised of as many as 1000 distinct neurologically active peptides [1]. These small disulfide rich peptide toxins are referred to as conotoxins (CTxs). There are approximately 700 *Conus* species, resulting in the staggering potential that 70,000–100,000 unique CTxs exist [1,2,3]. CTxs vary in their mechanism of action, and many have been found to modulate ion channels. These include α-CTxs which inhibit ligand gated nicotinic acetylcholine receptors (nAChRs), δ-CTxs which inhibit inactivation of voltage-dependent sodium channels, ω-CTxs which inhibit N-type voltage-dependent calcium channels, and κ-CTxs which inhibit potassium channels [2]. CTxs have a remarkable diversity of pharmacological functions, which provides valuable insight for the development of unique ligands for laboratory and therapeutic applications. Because CTxs represent extremely specific molecular probes, they are routinely used by researchers as a tool to study and differentiate between closely related biological receptor subtypes. The specificity and potency of CTxs that leads to their utility in the laboratory and their therapeutic potential also makes them potentially lethal if mishandled. The U.S. Army has calculated the LD_50_ of CTxs to be as low as 5 μg/kg [4,5]. For this reason, certain α-CTxs have been designated as Biological Select Agents or Toxins (Select Agents) by the U.S. Department of Health and Human Services [6]. Systematic investigation into methods of inactivation of α-CTxs is required in order to establish safe laboratory practices that ensure the health and well-being of researchers using these hazardous materials.

The current study is limited to α-CTxs. A survey of literature accessible in the National Center for Biotechnology Information (NCBI) pubmed resource shows that 74% (3385 of the 4564) of published works on CTxs are studies involving α-CTxs. The methods for safely decontaminating and neutralizing α-CTx waste vary greatly in universities where CTx research is conducted; this study evaluated currently employed biosafety protocols used in university research laboratories. Several current decontamination protocols for CTxs rely on procedures demonstrated to be effective on low molecular weight toxins of biological origin [7,8,9]. Other protocols use reactive disinfectants such as glutaraldehyde and/or formaldehyde; however, this treatment typically requires hazardous waste disposal of the resulting material. Some biosafety protocols exploit the susceptibility of CTxs to treatment with reducing agents, like dithiothreitol or β-mercaptoethanol, followed by thiol alkylation [10]. However, these decontamination protocols require specific reaction conditions to be effective, e.g., prolonged incubation at elevated temperatures or protection from light, making these methods difficult to scale-up for releases of large amounts of CTx, or decontamination of large surface areas. Destruction of the CTxs through the hydrolytic activity of concentrated (5–10 M) acids or bases is also impractical to safely scale-up to remedy large-scale spills [11]. Therefore, a rigorous study of a wide array of decontamination techniques was undertaken to determine the best method to safely and effectively mitigate the toxic hazard presented by α-CTxs as typically used in a practical research laboratory setting. The purpose of this study was to establish a decontamination protocol to efficiently disrupt the α-helical content and digest α-CTx by chemical treatment. To this end, the effects of various classes of chemicals, including oxidizing agents, reducing agents, crosslinking agents, and Contrex™ EZ, a commercially marketed protease enzyme-detergent mixture, were studied for their impact on the secondary and primary structure of α-CTx using circular dichroism (CD) spectroscopy and mass spectrometry (MS), respectively. Several α-CTxs were selected for this study due to their sequence similarity and disulfide connectivity to CTxs designated as Select Agents by the U.S. Department of Health and Human Services. The sequence of the α-CTxs used in this study and the U.S. Department of Health and Human Services definition for the primary sequence of a CTx select agent are shown in Table 1.

## 2. Results

### 2.1. Secondary Structure Analysis of Treated Conotoxin

**Figure 1:**
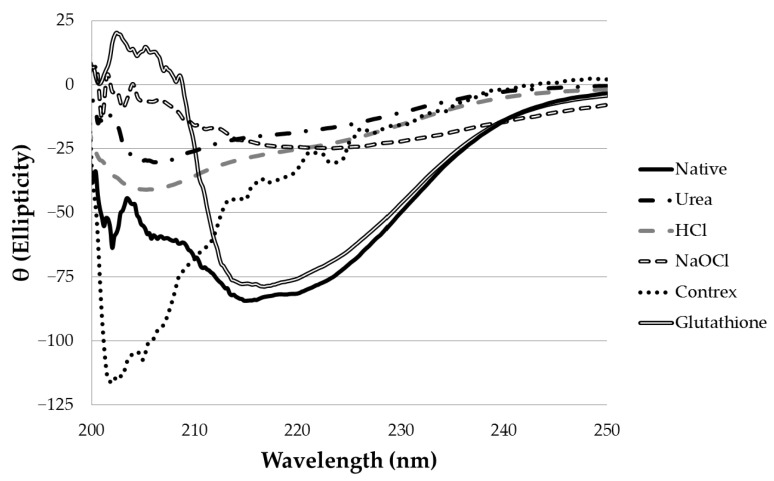
CD spectra for native α-CTx MII (L10V, E11A) and α-CTx MII (L10V, E11A) following chemical treatment for 15 min with 8 M urea, 6 M HCl, 6% sodium hypochlorite, 1% Contrex™ EZ, and 500 μM glutathione.

To determine the effect of chemical treatment on the secondary structure of α-CTx MII (L10V, E11A), α-helical content was estimated from the measured ellipticity at 222 nm. Typical CD spectra for chemical treatments of α-CTx MII (L10V, E11A) are shown in Figure 1.

The ability of chemical agents to disrupt the secondary structure of α-CTx MII (L10V, E11A) is summarized in Table 2. Untreated α-CTx MII (L10V, E11A) was estimated to have an α-helical content of 43.8 ± 2.3%; this is in close agreement with previously reported values for wild type and mutant α-CTx MII [16]. Based on CD analysis of treated peptides, the most-efficient chemical treatments for disrupting the secondary structure of α-CTx MII (L10V, E11A) were 8 M urea (91.2% decrease), 6 M HCl (81.5% decrease), 6% (*m*/*v*) sodium hypochlorite (80.5% decrease), and 1% (*m*/*v*) Contrex™ EZ (76.8% decrease). Chemical crosslinking with 2% (*m*/*v*) glutaraldehyde did not decrease the α-helical content, but interestingly, treatment with a combination of glutaraldehyde and formaldehyde seemed to disrupt secondary structure, with a 33.5% decrease in α-helical content. Treatment with ozone, hydrogen peroxide, and dithiothreitol (DTT) resulted in no significant change in α-helical content, and glutathione was only modestly effective at disrupting secondary structure, with a demonstrated decrease of 9.4%.

### 2.2. Primary Structure Analysis of Treated Conotoxin

**Figure 2:**
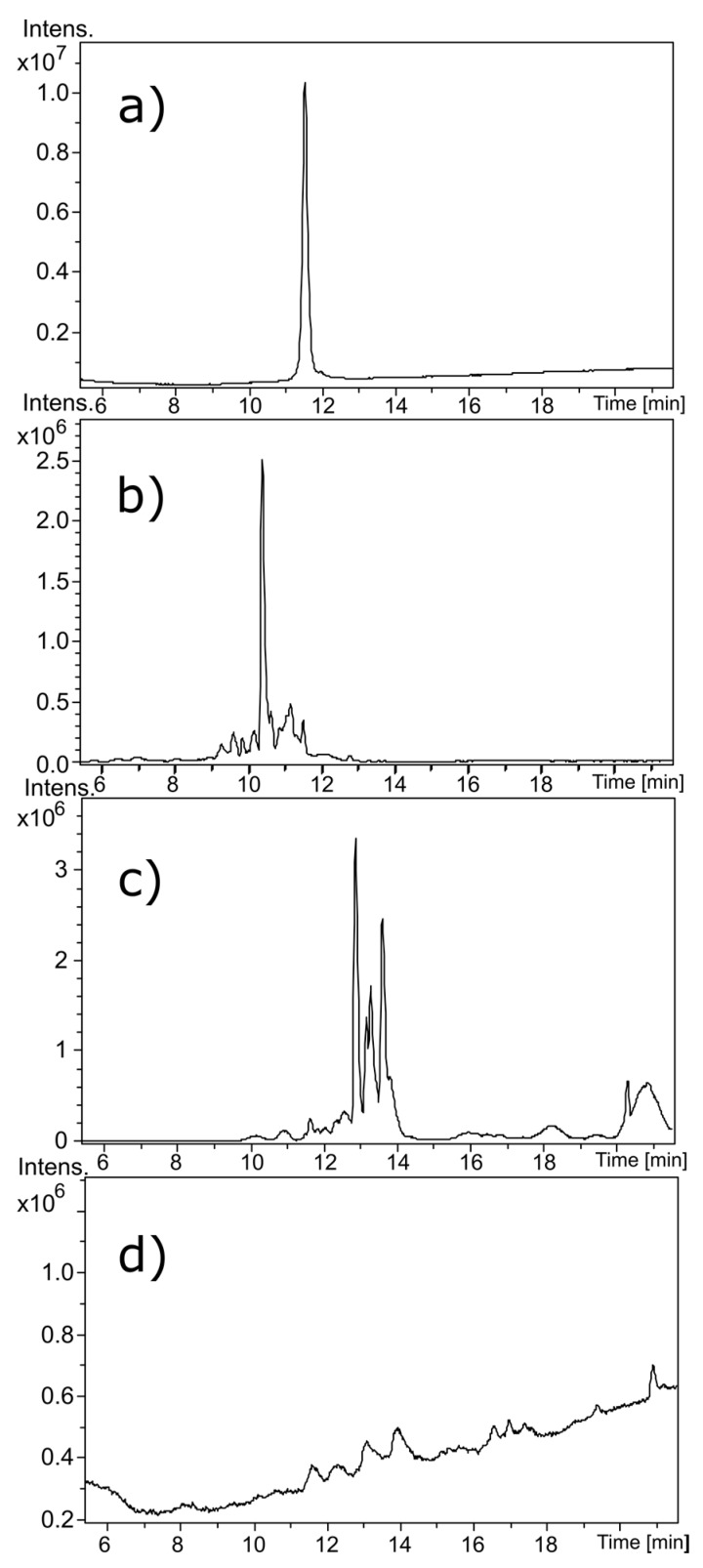
Liquid chromatography chromatograms of (**a**) native α-CTx MII (L10V, E11A); (**b**) Contrex ™ EZ treated α-CTx MII (L10V, E11A); (**c**) formaldehyde/glutaraldehyde treated; and (**d**) sodium hypochlorite treated.

**Figure 3:**
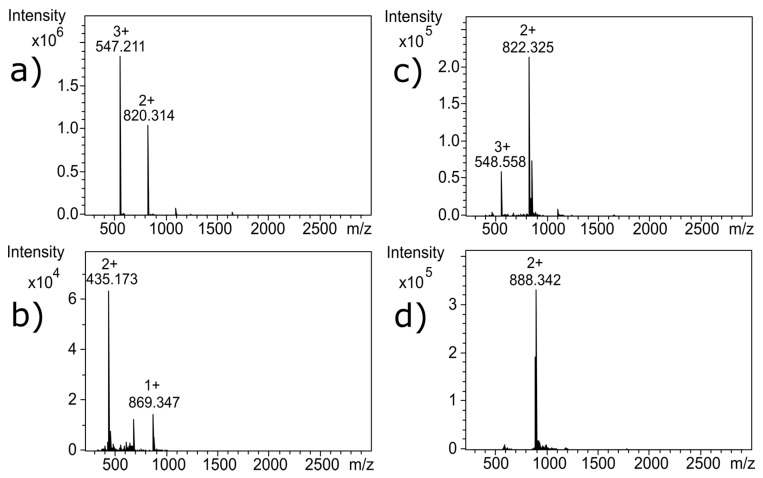
MS data for various α-CTx MII (L10V, E11A) samples where: (**a**) untreated α-CTx MII (L10V, E11A) measured by direct infusion; and (**b**) Contrex™ EZ treated samples in which digested peptide fragments of reduced MW are observed by LC-MS (The spectrum corresponds to the major peak observed in Figure 2b with a retention time 10.4 min.); (**c**) DTT treated sample measured by direct infusion with a mass shift of 4 Da, observed as a 2 Da shift from 820.314 to 822.325 in the doubly charged ion, indicating that disulfide bonds were reduced; and (**d**) formaldehyde/glutaraldehyde treated samples in which covalent modifications increase the MW of α-CTx MII (L10V, E11A), as observed by an increase from *m*/*z* 820.314 to 888.342 in the doubly charged ion. The spectrum corresponds to the major peak observed in Figure 2c with a retention time 12.9 min.

Figure 2 shows liquid chromatography (LC) chromatograms of untreated α-CTx MII (L10V, E11A) (a), and α-CTx MII (L10V, E11A) following treatment with Contrex™ EZ (b), formaldehyde/glutaraldehyde (c), and sodium hypochlorite (d). The molecular weight observed for untreated α-CTx MII (L10V, E11A) was 1639.618 Da, with a doubly charged ion observed at 820.314 *m*/*z* and a triply charged ion observed at 547.211 *m*/*z* (Figure 3a). Contrex™ EZ treatment resulted in digested peptide fragments of diminished molecular weights, including ions observed at 982.378 *m*/*z*, 883.308 *m*/*z* and a singly charged ion of 869.341 *m*/*z* shown in Figure 3b. Formaldehyde and glutaraldehyde are aggressive carbonyl reagents that react with proteins at primary amines. Glutaraldehyde alone, or in combination with formaldehyde, is commonly used as a disinfectant. Formaldehyde/glutaraldehyde treatment provided a modest increase in observed molecular weight to 1775.673 Da, supported by the increase from *m*/*z* 820.314 to 888.342 in the doubly charged ion (Figure 3d). The shift in *m*/*z* from 820.314 to 888.342 in the doubly charged ion corresponds to a mass shift of 136 Da in α-CTx MII (L10V, E11A). This result is consistent with the formation of imine bonds by two glutaraldehyde molecules forming intra-peptide crosslinks. The mass addition of each glutaraldehyde crosslink (C_5_H_8_) is 68 Da. The mass increase of 136 Da in the doubly charged mass spectrum of glutaraldehyde treated peptide can be attributed to the addition of C_10_H_16_ to the molecular formula of α-CTx MII (L10V, E11A). Inter-peptide crosslinking between several α-CTx MII (L10V, E11A) molecules was not observed. Sodium hypochlorite treatment entirely digested α-CTx MII (L10V, E11A), and no discernable peaks in the mass range 250–2900 Da were observed in the mass spectrum.

## 3. Discussion

Near-UV CD spectroscopy indicated that DTT effectively reduced the disulfide bonds of α-CTx MII (L10V, E11A), as shown by the disappearance in the band around 270 nm (Supplemental Figure S1). The near UV absorption of the disulfide bond occurs near 260 nm and is generally quite weak [17,18]. Reduction of the disulfide bonds was confirmed by MS as shown by the 4 Da shift in Figure 3c; the 4 Da shift is observed as a 2 Da shift from 820.314 to 822.325 in the doubly charged ion, compared to *m*/*z* for the untreated α-CTx MII (L10V, E11A) (Figure 3a). Interestingly, reduction of the disulfide framework did not disrupt the secondary structure of the peptide, and would therefore not be expected to guarantee inactivation of biological potency. If the CTx retains native structure, despite reduced disulfide bonds, the activity of the toxin must be assumed to be intact.

Ozonolysis of peptides has been shown to affect amino acids with aromatic side chains and histidine [19]. α-CTx MII (L10V, E11A) contains two histidine residues, and exposure to ozone for 24 h resulted in a mass shift of 16 Da in ~30% of the peptides (Supplemental Figure S2). Treatment with O_3_ did not affect the secondary structure of α-CTx MII (L10V, E11A), leading to the conclusion that ozonolysis is an ineffective decontamination method. Hydrogen peroxide treatment followed by exposure to ultraviolet light generates hydroxyl radicals with potential to cleave peptide bonds. However, hydrogen peroxide treatment did not alter the secondary or primary structure of α-CTx MII (L10V, E11A), as evidenced by the absence of either significant decrease in α-helical content by CD, or mass shift by MS (data not shown).

Treatment of α-CTx MII (L10V, E11A) with 8 M urea was effective at disrupting the secondary structure. The peptide samples treated with 8 M urea were diluted immediately prior to CD measurement in order to obtain each spectrum. Although treatment with urea did disrupt secondary structure, it did not alter the primary structure of α-CTx MII (L10V, E11A), as determined by MS. To be an effective decontamination method, additional treatment following urea induced peptide denaturation would be required. Treatment with 6 M hydrochloric acid was effective at denaturing α-CTx MII (L10V, E11A), but this is not a practical protocol for ameliorating spills due to highly hazardous properties.

Two chemical treatment options, sodium hypochlorite and Contrex™ EZ, were identified as the most effective and applicable laboratory decontamination methods reviewed in this investigation. Sodium hypochlorite (6% (*m*/*v*)) treatment caused complete disruption of secondary structure and digestion of α-CTx MII (L10V, E11A). This result may be due to excess sodium hydroxide present in commercial Clorox used to stabilize the sodium hypochlorite in the product, or because of oxidative cleavage of peptide bonds by sodium hypochlorite. Sodium hypochlorite has a pungent odor and acts as a strong oxidizer that can react unfavorably with common laboratory chemicals, however, bleach is ubiquitously available, inexpensive, and frequently used as a disinfectant in laboratories. Sodium hypochlorite (6% (*m*/*v*)) appears to be an effective method to use in biosafety protocols for the decontamination of α-CTxs in research laboratories.

**Figure 4:**
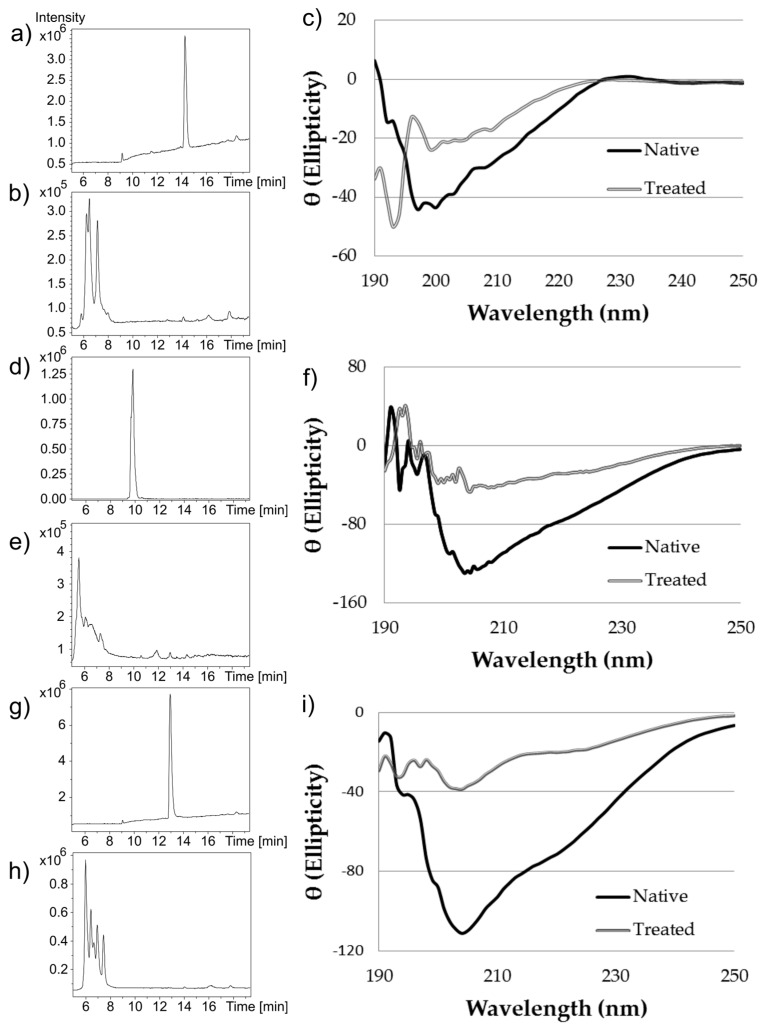
LC-MS chromatograms of (**a**) native α-CTx ImI; (**b**) Contrex™ EZ treated α-CTx ImI; (**d**) native α-CTx LvIA; (**e**) Contrex™ EZ treated α-CTx LvIA; (**g**) native α-CTx PeIA and Contrex™ EZ treated α-CTx PeIA (**h**). CD spectra are also shown for native and treated α-CTx ImI (**c**); α-CTx LvIA (**f**); and α-CTx PeIA (**i**). LC-MS data demonstrate the efficacy of Contrex™ EZ at eliminating the native α-CTx, and CD spectra confirm that Contrex™ EZ effectively disrupts the secondary structure of the α-CTxs.

For α-CTx decontamination, Contrex™ EZ lacks the negative characteristics of bleach, but retains desired activity. Contrex™ EZ contains an anionic surfactant detergent as well as Esperase, a non-specific endo-peptidase that has broad specificity and high activity in pH range 8–12 [20,21]. Thus, we sought to explore how robust chemical treatment of α-CTx with Contrex™ EZ was using α-CTxs PeIA, LvIA, and ImI. The result was consistent with Contrex™ EZ treatment of α-CTx MII (L10V, E11A) as confirmed by CD spectroscopy and MS (Figure 4). The α-helical content for α-CTx PeIA was observed to decrease from 36.6 ± 3.3% in native peptide, to 11.1 ± 7.7% in the Contrex™ EZ treated sample. Similarly, the α-helical content for α-CTx LvIA decreased from 37.4 ± 2.1% in the native peptide, to 10.1 ± 0.1% in the Contrex™ EZ treated sample. α-CTx ImI does not have the typical α-helical region found in the other α-CTxs used in this study, so the α-helical content was not calculated for this sample. However, the CD spectrum of α-CTx ImI did indicate a change in secondary structure upon treatment with Contrex™ EZ.

The secondary structure of each Contrex™ EZ treated α-CTx was shown to be disrupted, but not destroyed, and digestion produced peptide fragments of reduced molecular weight by mass spectrometry, not complete digestion. It is possible for miniaturized or partially digested peptide to maintain undesirable activity. Assessment of partially digested peptide activity was not evaluated. It is thus recommended that 6% sodium hypochlorite be used when handling highly potent α-CTxs to ensure complete digestion.

The results of this study indicate the use of 1% Contrex™ EZ (*m*/*v*) solution may be suitable for inclusion in biosafety protocols requiring decontamination of dilute solutions of α-CTxpeptides (e.g., HPLC waste, peptide synthesizer waste, filtrate, etc.), laboratory spills of dilute peptides, or cleaning of equipment exposed to trace amounts of α-CTxs. In situations requiring decontamination of concentrated solution or solid peptide, or highly potent α-CTx (e.g., select agent α-CTx), 6% sodium hypochlorite has been confirmed to be the recommended method to ensure inactivation. The scope of this study was limited to the evaluation of decontamination procedures for α-CTxs. The recommended decontamination procedures may not apply to all CTxs, particularly those with significant differences in disulfide framework, solubility, or peptide size.

In laboratories where use of bleach is acceptable, sodium hypochlorite 6% (*m*/*v*) solution proved to be an effective chemical treatment for α-CTxs. Alternatively, treatment with 1% solution of Contrex™ EZ resulted in a 76.8% decrease in α-helical content and digestion of α-CTx MII (L10V, E11A) into smaller peptide fragments as demonstrated by mass spectrometry. The results of this investigation indicate that partial digestion occurs within the first 15 min of treatment. For liquid chemical waste decontamination, all liquid waste containing α-CTxs, including HPLC waste, may be stored in 4 L carboys, to which 40 g of Contrex™ EZ powder should be added to achieve ~1% solution (% *w*/*v*). A 4 lb container of Contrex™ EZ can be purchased from fishersci.com for $54.00 USD, and 121 oz of Clorox HE performance bleach can be purchased from Amazon.com for $15.75 USD. Decontamination of a 4 L bottle of waste containing 100 μg conotoxin for every 1 mL of solution will cost on the order of $1.20 USD for Contrex EZ as compared to $1.60 USD for bleach. In the studies reported herein, the decontamination methods were tested on α-CTx containing solutions of high peptide content (1.7 mg of α-CTx MII (L10V, E11A) per 1.0 mL) and low chemical treatment concentration (1% Contrex™ EZ solution). A recommended time for decontamination of liquid waste is a minimum of one hour to ensure more than adequate time for α-CTx degradation.

## 4. Materials and Methods

### 4.1. Solvents and Reagents

Chemical treatments were prepared from 8 M urea, 30% hydrogen peroxide (*m*/*v*), 12 M hydrochloric acid (HCl), 50% glutaraldehyde (*m*/*v*), 37% formaldehyde (*m*/*v*), dithiothreitol (DTT), and glutathione purchased from Fisher Scientific (Pittsburgh, PA, USA). Contrex™ EZ powdered enzymatic detergent was purchased from Decon Labs (King of Prussia, PA, USA). Household Clorox bleach was used for the 6% sodium hypochlorite. High pressure liquid chromatography (HPLC) mobile phase consisted of 18 MΩ H_2_O and HPLC grade formic acid and acetonitrile (>99% purity, Fisher Scientific). All α-CTxs used in this study were purchased from CS Bio (Menlo Park, CA, USA) at a purity of >95%.

### 4.2. Chemical Treatments

The following treatment conditions were used: 8 M urea, 6% sodium hypochlorite, 1% Contrex™ EZ solution (*m*/*v*), 10% hydrogen peroxide (*m*/*v*) with UV irradiation, gaseous ozone, 6 M hydrochloric acid, 2% glutaraldehyde (*m*/*v*), 1% formaldehyde (*m*/*v*)/1% glutaraldehyde (*m*/*v*), 10 mM dithiothreitol, and 500 μM glutathione. For each of these treatments, the reaction was allowed to proceed for 15 min prior to sample dilution and analysis of secondary structure using CD spectroscopy. Prior to analysis by HPLC-MS, Contrex™ EZ, the reaction was quenched and samples were purified using a Pierce Strong Cation Exchange Spin Column (Thermo Fisher Scientific, Waltham, MA, USA), a wash buffer composed of 25 mM sodium acetate, pH 5.5, and an elution buffer of 25 mM sodium acetate, 1 M NaCl, at pH 5.5. All other reactions were quenched and samples were purified using Pierce PepClean™ C_18_ Spin Columns (Thermo Fisher Scientific, Waltham, MA, USA) activated with 50% methanol (*v*/*v*), and a wash buffer composed of 5% acetonitrile (*v*/*v*) and 0.5% trifluoroacetic acid (*v*/*v*), and an elution buffer of 70% acetonitrile (*v*/*v*). Ozone gas was generated with a Villa 1000 ozone generator from SD International, Inc. (Tallahassee, FL, USA) operated at an output of 1800 mg ozone per hour. The reaction was performed with a 50 μM solution of α-CTx MII (L10V, E11A) exposed to flowing ozone captured in a custom-made reaction vessel for up to 24 h. α-CTx MII (L10V, E11A) treated with 10% hydrogen peroxide was exposed to UV irradiation in a biosafety hood with a UV lamp operating at an intensity of 125 μW/cm^2^ at a distance of ~1 m for 20 min.

### 4.3. Circular Dichroism Spectroscopy

CD spectra were recorded on a Jasco J-810 spectropolarimeter (Jasco, Inc., Easton, MD, USA) with a cell path length of 1 cm at room temperature in a nitrogen atmosphere. Scans were acquired from 190 to 250 nm and 250 to 300 nm for far-UV and near-UV, respectively. The bandwidth of 1 nm, a speed of 50 nm/min, and a resolution of 0.5 nm were used. A total of 5 scans per sample were averaged, baselines were subtracted, and each sample was run in triplicate. A final concentration of 50 μM and 500 μM were used for far-UV and near-UV scanning wavelengths, respectively. Analysis and processing of data were carried out with the Jasco system software and Microsoft Excel. Mean residue ellipticity (θMRE, in deg × cm^2^ × dmol^−1^) for each spectrum was calculated from the formula θMRE = θ/(10 Crl), where θ is the measured ellipticity in millidegrees, Cr is the molar concentration, and l is the path length in centimeters. The α-helical content was estimated from the formula θMRE = −30300fH − 2340, where fΗ is the fraction of α-helical content (fΗ × 100, expressed as a percentage) calculated from the θMRE at 222 nm, is a widely used proxy for helical secondary structure that is useful to assess disruption of the secondary structure, as is performed here [22]. The final concentration of the chemical treatments varied and were determined individually as the maximum concentration at which a suitable signal at 222 nm could be obtained without noise affecting the reliability of the measured signal. The final concentration of each chemical treatment at the time it was measured is summarized in Supplemental Table S1.

### 4.4. High Performance Liquid Chromatography-Mass Spectrometry

Untreated and treated α-CTxs were analyzed by HPLC-MS using an ultra-high resolution Quadrupole Time of Flight (QTOF) instrument (Bruker maXis, Bruker Corporation, Billerica, MA, USA) by direct injection to the MS or by HPLC-MS. The electrospray ionization (ESI) source was operated under the following conditions: positive ion mode; nebulizer pressure: 0.4 or 1.2 Bar; flow rate of drying gas (N_2_): 4 or 8 L/min; drying gas temperature: 200 °C; voltage between HV capillary and HV end-plate offset: 3000 V to −500 V; mass range was set from 250 to 2900 *m*/*z*; and the quadrupole ion energy was 4.0 eV. Low concentration ESI tuning mix (Agilent Technologies, Santa Clara, CA, USA) was used to calibrate the system in the mass range. Samples analyzed by direct infusion with a syringe pump at a flow rate of 240 μL/h. HPLC separation was achieved using a Dionex UltiMate^®^ 3000 uHPLC system (Dionex Corporation, Sunnyvale, CA, USA) equipped with a Thermo Acclaim™ 120 C_18_ column (2.1 × 150 mm, 3 μm) (Thermo Fisher Scientific, Waltham, MA, USA). The mobile phase was 0.1% formic acid in water (Buffer A) and acetonitrile (Buffer B) with a flow rate of 0.3 mL/min. A linear gradient method was used to separate the mixture starting at 5% acetonitrile and ending at 65% acetonitrile over 25 mi. The sample injection volume was 1 μL. Data were analyzed using the Compass Data Analysis software package (Bruker Corporation, Billerica, MA, USA).

## Figures and Tables

**Table 1:** Amino acid sequences for the α-CTxs used in this study and CTx select agent sequence motif. For the α-CTxs in Table 1 all cysteine residues are present as cystines with the 1st and 3rd cysteine and the 2nd and 4th cysteine joined through disulfide bonds. For the select agent, the X_N_ amino acid represents the following: X_1_ may be any or no residue; X_2_ is N or H; X_3_ is R or K; X_4_ is N, H, K, R, Y, F or W; X_5_ is Y, F or W; X_6_ is S, T, E, D, N, or Q; and X_7_ is any or no residue. Emboldened residues indicate conserved elements of the select agent motif.

Name	Sequence	Ref.
LvIA	G **C C** S H **P** A **C** N V D H P E I **C**	[12]
MII (L10V, E11A)	G **C C** S N **P** V **C** H V A H S N L **C**	[13]
ImI	G **C C** S D **P** R **C** A W R - - - - **C**	[14]
PeIA	G **C C** S H **P** A **C** S V N H P E L **C**	[15]
Select Agent	X_1_ **C C** X_2_ **P** A **C** G X_3_ X_4_ X_5_ X_6_ - **C** X_7_	[6]

**Table 2:** Summary of the estimated α-helical content of α-CTx MII (L10V, E11A) prior to and following chemical treatment at 15 min reaction time. Samples were run in triplicate with the average ± standard deviation shown. The α-helical content represents the percent decrease as compared to the control peptide, which has α-helical content of 43.8 ± 2.3%.

Treatment	α-Helical Content	Δ α-Helical Content
No Treatment	43.8 ± 2.3%	NA
8 M Urea	3.9 ± 0.7%	91.2%
6 M Hydrochloric Acid	8.1 ± 0.4%	81.5%
6% Sodium Hypochlorite	8.6 ± 0.4%	80.5%
1% Contrex™ EZ	10.2 ± 1.8%	76.8%
1% Glutaraldehyde/1% Formaldehyde	29.1 ± 2.3%	33.5%
500 μM Glutathione	39.6 ± 1.4%	9.4%
10 mM Dithiothreitol	43.5 ± 4.8%	No Change
2% Glutaraldehyde	44.1 ± 1.1%	No Change
Ozone	46.7 ± 2.5%	No Change
10% Hydrogen Peroxide/UV	47.0 ± 7.0%	No Change

## Supplementary Materials

The following are available online at www.mdpi.com/2072-6651/9/9/281/s1. Figure S1: Near UV data of untreated and DTT treated α-CTx MII (L10V, E11A) is shown. Figure S2: α-CTx MII (L10V, E11A) exposed to ozone for 24 h. Table S1: Concentration of chemicals when analyzed by far-UV CD spectroscopy.
