# Supplementary material for: α-Conotoxin Decontamination Protocol Evaluation: What Works and What Doesn’t
Source: Toxins (Basel). 2017 Sep 14;9(9):281. doi: 10.3390/toxins9090281 (PMC5618214; doi:10.3390/toxins9090281)
Supplement: Supplementary file 1 [file toxins-09-00281-s001.pdf]

Supplementary Materials: α-Conotoxin Decontamination Protocol Evaluation: What Works and What Doesn't

Matthew W. Turner, John R. Cort and Owen M. McDougal

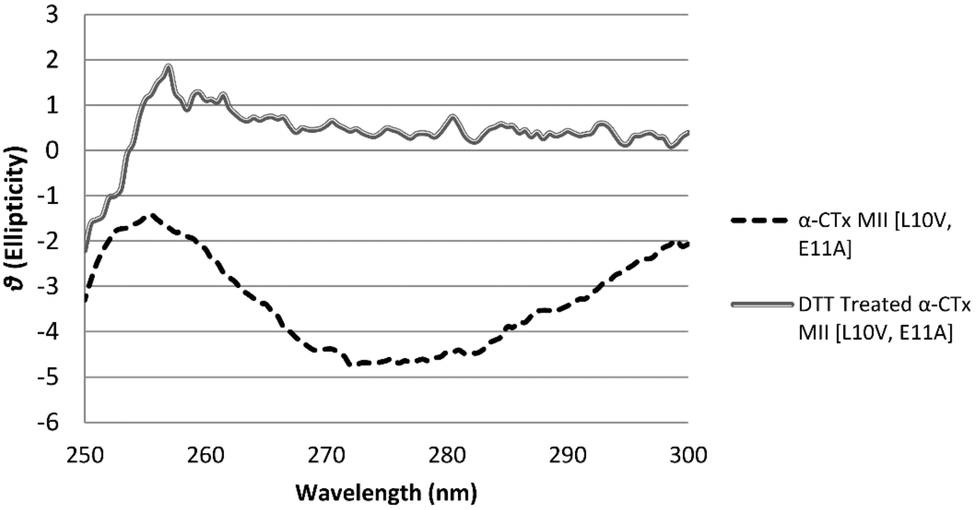

Figure S1. Near UV data of untreated and dithiothreitol treated α-CTx MII (L10V, E11A) is shown. The loss of the broad band at 275 nm corresponds to the reduction of disulfide bonds.

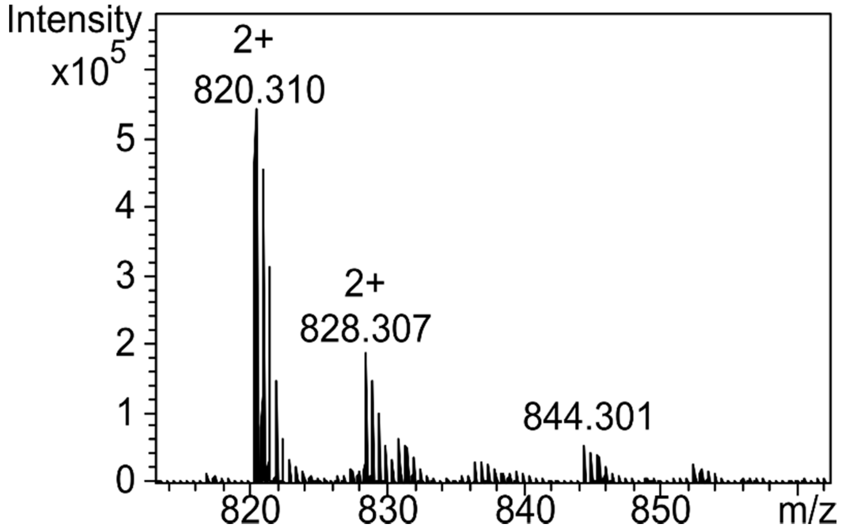

Figure S2. α-CTx MII (L10V, E11A) exposed to ozone for 24 hrs. Approximately 30% of α-CTx MII (L10V, E11A) has a mass shift of 16 Da following ozone treatment, as observed as 8 Da shift from 820.310 to 828.307 in the doubly charged ion. This did not result in a change in secondary structure.

Table S1. Concentration of chemicals when analyzed by far-UV CD spectroscopy. The concentration of α-CTx MII (L10V, E11A) was 50 μM in each trial.

**Treatment**	**Concentration**
6% Sodium hypochlorite	0.3%
1% Contrex ™ EZ	0.05%
8 M Urea	80 mM
6 M HCl	60 mM
2% Gluteraldehyde	0.5%
1%Gluteraldehyde/1% formaldehyde	0.5%/0.5%
10 mM DTT	500 μM
500 μM Glutathione	500 μM
10% Hydrogen peroxide	0.03%
